# Part II, Provider perspectives: should patients be activated to request evidence-based medicine? a qualitative study of the VA project to implement diuretics (VAPID)

**DOI:** 10.1186/1748-5908-5-24

**Published:** 2010-03-18

**Authors:** Colin D Buzza, Monica B Williams, Mark W Vander Weg, Alan J Christensen, Peter J Kaboli, Heather Schacht Reisinger

**Affiliations:** 1 The Center for Research in the Implementation of Innovative Strategies in Practice (CRIISP), Iowa City VA Medical Center, 601 Highway 6 West, Mail Stop 152, Iowa City, IA, 52246-2208, USA; 2 Division of General Internal Medicine, Department of Internal Medicine, University of Iowa Carver College of Medicine, Iowa City, IA, USA; 3 Department of Psychology, University of Iowa, Iowa City, IA, USA

## Abstract

**Background:**

Hypertension guidelines recommend the use of thiazide diuretics as first-line therapy for uncomplicated hypertension, yet diuretics are under-prescribed, and hypertension is frequently inadequately treated. This qualitative evaluation of provider attitudes follows a randomized controlled trial of a patient activation strategy in which hypertensive patients received letters and incentives to discuss thiazides with their provider. The strategy prompted high discussion rates and enhanced thiazide-prescribing rates. Our objective was to interview providers to understand the effectiveness and acceptability of the intervention from their perspective, as well as the suitability of patient activation for more widespread guideline implementation.

**Methods:**

Semi-structured phone interviews were conducted with 21 primary care providers. Interviews were transcribed verbatim and reviewed by the interviewer before being analyzed for content. Interviews were coded, and relevant themes and specific responses were identified, grouped, and compared.

**Results:**

Of the 21 providers interviewed, 20 (95%) had a positive opinion of the intervention, and 18 of 20 (90%) thought the strategy was suitable for wider use. In explaining their opinions of the intervention, many providers discussed a positive effect on treatment, but they more often focused on the process of patient activation itself, describing how the intervention facilitated discussions by informing patients and making them more pro-active. Regarding effectiveness, providers suggested the intervention worked like a reminder, highlighted oversights, or changed their approach to hypertension management. Many providers also explained that the intervention 'aligned' patients' objectives with theirs, or made patients more likely to accept a change in medications. Negative aspects were mentioned infrequently, but concerns about the use of financial incentives were most common. Relevant barriers to initiating thiazide treatment included a hesitancy to switch medications if the patient was at or near goal blood pressure on a different anti-hypertensive.

**Conclusions:**

Patient activation was acceptable to providers as a guideline implementation strategy, with considerable value placed on the activation process itself. By 'aligning' patients' objectives with those of their providers, this process also facilitated part of the effectiveness of the intervention. Patient activation shows promise for wider use as an implementation strategy, and should be tested in other areas of evidence-based medicine.

**Trial registration:**

National Clinical Trial Registry number NCT00265538

## Background

Hypertension affects more than 65 million Americans and more than 1 million veterans in the Veterans Administration (VA) [1,2]. Despite recent improvements in the detection and management of high blood pressure, studies suggest hypertension is still poorly controlled in at least half of VA patients, and likely more in other settings [1,3-6]. Guidelines suggest thiazide diuretics should be given as first-line therapy for uncomplicated hypertension and more frequently added to intensify existing regimens, but thiazides are under-utilized, and identification and appropriate treatment of patients with hypertension remains inadequate [4-8]. This 'quality gap' between evidence-based guidelines and clinical management of hypertension is not simply a matter of provider knowledge, but may be more attributable to clinical inertia (*i.e*., failure to initiate or intensify therapy when indicated), among other possible factors [5,9-11].

Provider-targeted interventions that aim to close this 'quality gap' in hypertension management have demonstrated mixed success. Provider education strategies and audit-and-feedback interventions have had little effect on management or control [12-14], while computerized reminders have shown inconsistent results [13,15-17]. However, interventions that incorporate someone other than the provider (*e.g*., pharmacist, nurse) into managing the patient's hypertension have shown more promise in supporting guideline-concordant treatment decisions [18]. The potential role of patients in supporting such evidence-based care is less explored.

Patient-targeted hypertension interventions have usually aimed to modify lifestyle risk factors or improve treatment adherence, and not alter clinical decision-making. However, patient education has been shown to enhance the success of some provider- or institutionally-targeted hypertension management interventions when provided in concert [12,13,18], and evidence from other areas of care suggests providing patients with evidence-based educational materials in clinics may assist providers in justifying evidence-based treatment decisions [19,20]. The study reported here follows an intervention that aimed to support guideline-concordant treatment not simply by educating, but by specifically 'activating' patients to engage their providers and request evidence-based therapy.

'Patient activation' uses the techniques of social marketing and direct-to-consumer (DTC) advertising to motivate patients to undertake a suggested action [21]. For example, printed materials may be designed to educate patients with a chronic disease in a manner specifically focused on motivating exercise or self-management [22,23]. As a guideline implementation strategy, the techniques of patient activation have been attempted only on a limited basis, and while not rigorously evaluated, have thus far shown mixed success [22,24-26]. Our study follows what was, to our knowledge, the first randomized controlled trial (RCT) of a patient activation intervention to improve adherence to clinical practice guidelines. In this trial, patients were provided with tailored information about their blood pressure, including risks and appropriate therapy, framed as motivation to pursue a suggested action: discussing the information with their providers. The intervention was successful in prompting both high patient-provider discussion rates and a significant increase in guideline-concordant prescribing [27].

While trial data show increased discussion and prescribing rates, the limitations of these measures and a paucity of similar research leaves unanswered questions concerning the process, acceptability and wider suitability of the intervention among providers:

1. What factors or elements of the intervention process facilitated or prevented changes in prescribing behavior? Which of these were unique to this intervention, or might be modifiable? Replication and future adaptation require an understanding of these factors and their context and consistency within the intervention, and failure to detect differences between implementation as planned and as practiced reduces the utility of outcome data [28].

2. How acceptable was the intervention to providers as stakeholders whose cooperation would be necessary for broader implementation? Evidence suggests implementation strategies may not be widely accepted or adopted by providers who feel their decision latitude is unnecessarily diminished [24,29-31], and DTC marketing is controversial [32,33]. What were provider attitudes towards this intervention that attempted to alter their decision-making by targeting the patient or 'consumer' directly, and how would they feel if it were implemented more broadly or applied to other aspects of care?

These questions were addressed through semi-structured interviews of participating primary care providers, complemented by patient perspectives reported in a companion article [34]. We report here results on: how the intervention created or facilitated changes in the prescribing behavior of participating providers; what barriers may have prevented changes in prescribing behavior; and how acceptable providers found the intervention strategy and its various components. From these and complementary patient results, we also hope to inform a broader understanding of the suitability of patient activation strategies to implement guidelines on a larger scale, for other therapies, and in alternate settings.

## Methods

### The intervention trial

This investigation was conducted following a RCT of a patient activation intervention to encourage patients with hypertension to speak with their provider about starting a thiazide diuretic [27]. All intervention patients received an individualized letter educating them about the risks of their hypertension, possible benefits of thiazides, and their current anti-hypertensive regimen, while also suggesting they discuss this information with their provider. The intervention included three arms: A, B, and C. Patients in arm A received only the letter, while patients in arm B also received the offer of twenty dollars for discussing the letter with their provider (regardless of whether or not a thiazide was prescribed), as well as a six-month co-pay reimbursement ($48) if prescribed a thiazide. Patients in arm C received the letter and financial incentive, as well as a phone call from a health educator to remind them of the letter and to answer any questions about the intervention. All patients were asked to return a postcard with their provider's signature, indicating whether thiazides were discussed and prescribed. Control patients received usual care. Control arms were divided into 'pure controls' and 'contaminated controls.' Pure controls were patients of randomly assigned providers who saw no patients who received the intervention letter. Contaminated controls were patients of providers who saw both patients who received intervention letters (intervention arm A, B, or C) and those who did not.

### Data collection

Telephone interviews were conducted with 21 providers who participated in the intervention at the Iowa City and Minneapolis Veterans Affairs Medical Centers (VAMCs) and four community-based outpatient clinics (CBOCs). The providers were purposefully sampled by site. To increase the likelihood they experienced the intervention, the sample also was limited to the 55 (30 from IA and 25 from MN) providers who had seen at least four intervention patients. From this sample, providers were randomly selected and emailed a formal request letter, followed by a reminder phone call after two weeks, if necessary. The recruitment process continued until data redundancy was reached, and approximately equal numbers were recruited from each site (n = 10 IA; n = 11 MN). In total, 41 providers were emailed. Of those, 13 providers did not respond to emails or phone calls, four declined, and three were unable to schedule time during the study period (Table 1). The study was approved by the Institutional Review Boards and Research and Development Committees at the Iowa City and Minneapolis VAMCs. Written consent was obtained with permission to record the interview.

**Table 1:** Providers response rate by facility type and title

	Total	Respondents	Non-respondents
Total	41	21 (51.22%)	20 (48.78%)

Facility Type			
VAMC		11 (26.83%)	10 (24.39%)
CBOC		10 (24.39%)	10 (24.39%)

Provider Type			
Physician (MD, DO)		15 (36.58%)	15 (36.58%)
Nurse Practitioner		3 (7.32%)	2 (4.88%)
Physician Assistant		3 (7.32%)	3 (7.32%)

Reason for non-response			
Declined		NA	4 (9.75%)
No Response		NA	13 (31.71%)
Unable to Schedule		NA	3 (7.32%)

All interviews were performed between May and September 2008 by two of the authors (CBD, HSR). A semi-structured interview guide was used, with open-ended and probing questions designed to elicit information relevant to effectiveness, acceptability, and wider applicability of the intervention, the main research questions for the qualitative provider sub-study (See Additional file 1). The interview guide was revised as new content was incorporated from previous interviews; however, the revisions of the interview guide primarily focused on clarification of questions and adding additional probes. Interviews lasted 20 to 37 minutes (median = 30.15) and were documented with a digital voice recorder. Recordings were transcribed verbatim by a trained research assistant, and carefully reviewed against the original recording by the interviewer. Subjects were identified in transcripts by randomly assigned numbers.

### Data analysis

Initial analysis of the first six transcripts was conducted by three study team members (CBD, HSR, MBW) who developed a coding template based upon the research objectives, interview guide, and interview content [35]. The coding template was used to conduct a thematic content analysis for all interviews, with content codes assigned to categorize passages [36,37]. The next three interviews were then independently coded for content themes to test the codebook. In cases where coders disagreed, differences were discussed until consensus was reached. Consensus involved the discussion of disagreements among interviewers, including where the coding of passages should stop and start, passages a coder did not mark, or the removal of a code from a particular passage. The consensus process served to increase the validity and reliability of the codebook by refining the content boundaries of the codes and making coding more consistent. The final consensus was then entered into NVivo 8, a software package for qualitative data management and analysis [38]. The remaining 21 total transcripts were content coded by the first author (CBD). Two coders (CB, MW) conducted matrix coding of passages categorized by thematic content to identify specific provider responses and the distribution of provider opinions [39]. For example, passages from each provider that were coded 'opinion of intervention' were independently classified by each coder into the discreet categories of positive, negative, neutral, or unknown; disagreements were adjudicated by a third coder (HSR) who acted as a tiebreaker.

## Results

### Intervention trial summary

The results from the intervention trial showed that, on average, 61% of intervention patients discussed thiazides with their providers [27]. In the three intervention arms, 26% of patients were prescribed a thiazide compared to only 6.7% of control patients. The addition of financial incentives and a phone call from a health educator each showed modest, incremental effects on discussion rates and subsequent thiazide prescribing.

Below, we focus on the results from the semi-structured provider interviews, which revealed a number of opinions and common themes that help to explain this demonstrated effectiveness and further speak to both the acceptability and wider applicability of the intervention.

### Typical consultations

Of the 21 participating providers, 15 were physicians, three were physician assistants, and three were nurse practitioners. All providers indicated they discussed hypertension and thiazides at the prompting of intervention patients. Conversations were initiated at varying times in the visit and were of varying length, although most providers indicated the conversation lasted five minutes or less. All providers thought most patients were comfortable initiating the conversation, although several pointed out that those patients that were not comfortable likely did not bring in the letter. Only one provider remembered that a patient specifically requested to be prescribed a thiazide, and most providers described their discussions as fitting with one or both of the following themes:

1. 'Should I be on this medication?' Many providers described discussions in which intervention patients produced the intervention letter or postcard and asked if they should be on a thiazide. This was typically described as a neutral question, although one provider indicated that one patient was alarmed there might be an oversight.

2. 'I was supposed to bring this to you [in order to get some money].' Many providers also described discussions in which intervention patients produced the intervention letter or postcard as a task they were instructed to complete. Providers also mentioned that some such patients brought up the incentive as a reward for completing the task.

### Influence on prescribing behavior

Most providers (19/21) prescribed thiazides to at least one patient as a result of the intervention. Their descriptions of the influence of the intervention can be broadly categorized into three themes: reinforced their existing knowledge or prescribing behavior, changed their approach to hypertension management, and patient activation itself lowered barriers to thiazide prescribing.

#### The intervention reinforced existing knowledge or prescribing behavior

More than half of interviewed providers suggested the effect of the intervention was not to change their clinical approach to hypertension management, but rather to reinforce their training and current prescribing practice in a number of ways. Some cited their clinical experience and understanding of the role of thiazides in suggesting the intervention simply 'acted like a reminder' to consider a thiazide. Others said the intervention brought their attention to specific patients for whom they would typically prescribe a thiazide, but were not on one:

'There were some that were oversight...they were supposed to be on hydrochlorothiazide. They have no reason not to be on it, and yet they...were not on it, and your letter brought my attention to it.'

A few providers explained they manage over 1,000 patients, so 'oversights' can happen, particularly with new patients or those co-managed with non-VA providers. Several providers elaborated on how the intervention brought the patients' treatment regimens under new scrutiny:

'With our co-managed patients...I just...tended to assume, you know, that a thiazide had been tried at some point, if they're already on something that I would've picked second, third, or fourth, you know, as an agent. And, and I've, I mean that was, uh, a big message to me that I can't assume that.'

Two providers also suggested the intervention provided previously unknown information that moved patients into a category for which the provider would usually prescribe a thiazide:

'Something that came up a couple times...the letter, it said 'on a certain date the blood pressure had been high,' and that date had been like on a specialty care visit, so it was a number that I probably...wasn't aware of...because maybe they were fine the day I saw them...and it did change my plan, you know, after...seeing that.'

#### The intervention changed the provider's approach to hypertension management

Several providers suggested the intervention didn't just reinforce existing knowledge or prescribing behavior, but actually changed their clinical approach to hypertension management. Some stated the intervention provided new information about thiazides, or otherwise changed their view of thiazides as a first-line management option:

'It helped certainly, you know, if you come up to me with a letter and said, 'hey, this evidence and all that, you can do this with less cost and equal efficacy,' then certainly, you know...that would change my...practice, behavior, certainly, yeah.'

Others emphasized the intervention brought their attention to patients who were not simply oversights, but for whom they may not have considered a thiazide:

'It was almost as if, uh, someone were looking over my shoulder and saying 'here, try this.' I think in most cases I agreed and incorporated that as one of the medications.'

#### Patient activation itself lowered barriers to thiazide prescribing

Many providers also described the process of patient activation as lowering barriers that might otherwise prevent prescribing a thiazide. Some suggested the intervention made patients more receptive to adding or switching to a thiazide. Particularly with co-managed patients, several providers said that patients 'that have been on...whatever [other] medication for years and years' would typically be hesitant to change, especially if their blood pressure was near or at goal. These providers suggested the intervention lowered a barrier to thiazide prescribing by providing patients with information and facilitating a discussion:

'Through...the discussion of them even receiving this invitation in, in the first place, uh, prompted them to be more willing to start the medicine.'

'Some of them didn't want to change, but...a couple of them said, 'well, let's, you know, with that information, let's change over'.'

Other providers described the intervention as 'aligning' patient and provider 'priorities':

'One of the most difficult...problems for a practicing, full-time clinician is trying to stay on schedule, and if we can help patients to have the same objectives, align our...priorities, then I think we'll reach them. Um, the problem often times is that there's another issue, a distracter issue that the patients want to talk about. They don't frequently want to talk about or mention a chronic asymptomatic disease. They have a rash on their elbow and a little ringing in their ear...and they'll often consume time just unloading their frustrations. If, on the other hand, there was an incentive for them to, uh, focus their energies on the same objectives WE have, then I think we could meet those objectives, but we have to stay on time.'

### Influence on prescribing behavior beyond the intervention

Over the course of the intervention, providers who had patients in the intervention were somewhat more likely to prescribe a thiazide to their patients in the control group (*i.e*., 'contaminated' controls) than the providers who had no intervention patients, but had control patients (*i.e*., 'pure' controls) (13.2% versus 5.7%; P = .09). Correspondingly, 11 of 17 providers stated they felt the intervention changed the way they prescribed to patients not involved in the study. Most providers said they were more likely to think of thiazides first when managing hypertensive patients, and some suggested it changed the question in their minds from 'what anti-hypertensive should be used?' or 'is the patient's hypertension controlled?' to 'why is this patient not on a thiazide?' Below is a sampling of responses to the question 'do you think it [the intervention] changed the way you prescribed thiazides with other patients?'

'I think it really re-emphasized to me, you know, going with thiazide diuretics as the first choice.'

'Yeah, it did...believe me. Uh, after I started getting that letter I started looking more closely at, uh, if I have a patient with hypertension now. Honestly, because of your letter I look at it, I look at *why is he not on hydrochlorothiazide*.' (emphasis added).

Providers who felt the intervention did not change their thiazide prescribing behavior beyond the intervention mostly emphasized that it was because they already prescribed thiazides regularly:

'I don't think it changed, I don't see how it could change...because I, uh, I like thiazides...I'm already a believer.'

### Barriers

Providers suggested a number of barriers to the influence of the intervention that are likely to restrict concordance with hypertension guidelines more generally. They can be categorized according to three common themes: guidelines are not universally applicable, reluctance to 'rock the boat', and cost and inconvenience.

#### Guidelines are not universally applicable

Some providers described the influence of the intervention--and guideline concordance more generally--as limited according to the characteristics of each particular patient:

'Each patient is individual...and...they need individual attention. And, uh, sometimes they fall into guidelines sometimes they don't. You know, for example, I have an eighty-five year old patient, uh, who has a blood pressure of 170, 180, and I cannot lower that to 140, patient becomes dizzy and light-headed, I cannot use the guidelines. So I have to accept higher blood pressure. You know, I have patients that they have supine hypertension. Their blood pressure is 200 when they lay down, when they stand up they're up to 120. And uh, every time they go to the hospital, their blood pressure is high. They put them on a bunch of blood pressure medications. They come out and they fall down...I cannot use the guideline for such [a] patient like that.'

Many other providers explained that, especially at the VA, they often see geriatric patients that are more likely to have multiple co-morbidities or contra-indications that make thiazides unsuitable or indicate a greater benefit from another anti-hypertensive:

'You know, my patients are older. They have prostate issues, and they go to bathroom too often, they have arthritis, they have difficulty to get to the bathroom...some they had problems with hypokalemia or renal issues that they were not a candidate for the medication...and, uh, my patients are diabetic, they have coronary artery disease, they have, you know, metabolic syndrome, so I think ACE inhibitors and ARBs are more selective for them than you know, just, uh, hydrochlorothiazide.'

#### Reluctance to 'rock the boat'

Many providers explained that, while they understand the benefit of a thiazide, they or often their patients were nevertheless hesitant to add or switch to a thiazide if the patient's blood pressure was already at or near goal. In the RCT, patients who were not controlled at the time of their primary care visit were 3.3 times more likely to be prescribed a thiazide than those who were controlled:

'I think [it] kind of depended where their blood pressure was at, you know, if their numbers were controlled without side effects on the regimen that they were on, I think there was, you know, a little bit of uh, um, kind of a sentiment on the part of the patient and maybe a little reluctance to kind of rock the boat.'

This was particularly an issue with new or co-managed patients:

'The...difficulty with being prescribed...are those patients that [have] been on...another medication for years by the previous provider or by their private...physician, and so it's hard for you to convince them to change to something different because they say 'Well I've been on this for like, ten years now and my blood pressure is controlled, why do you want to change it now?"

#### Cost and inconvenience

Several providers also mentioned cost and inconvenience to patients as a barrier. Some discussed patients for whom travel to their VA clinic was lengthy or difficult, so they didn't want to be switched if it required an extra visit for labs. Another provider explained that, although the co-pay at the VA is a flat eight dollars for each medication, patients often have many prescriptions, so the cost of adding one more can be prohibitive. Based on a similar rationale, another provider described looking to other anti-hypertensives with a broader range of indications, thus possibly eliminating the need for another prescription:

'Diuretics, like thiazide...sometimes I say 'why I should make this guy spend eight dollars?' Let me just give an ACE and get two things [hypertension and diabetes treatment] done.'

### Acceptability of the intervention

Almost all providers (20/21) had a positive opinion of the intervention strategy, but many expressed nuanced opinions, highlighting positive aspects and sometimes noting reservations.

#### Positives

When asked their opinion of the intervention, some providers discussed its positive effect on their approach to hypertension, but many more focused on the way it educated patients and facilitated discussion during the consultation. About one-third stated they had a positive opinion of the intervention at least in part because it prompted a positive change in their management of hypertension for some patients. About one-third of providers also expressed a favorable opinion of the intervention because it made patients more informed about their hypertension and different therapy options. Finally, most providers had a positive opinion of the intervention because it promoted among patients a greater interest and involvement in their hypertension management. These first three themes were often expressed in various combinations by providers:

'I really liked and, as I said it brought up, it made me think about things a little differently in some cases and it brought up great conversations with the patients.'

'I think it's good...it makes patients a little more pro-active about their healthcare...they were interested in it and it made them actually, you know, talk to you about their blood pressure.'

'I think it's a great idea...for many reasons. The actual subject matter, of course, is very pressing. Poorly-controlled hypertension is a well-recognized problem, and under-utilization of diuretics, and it's also um, a nice intervention to involve patients and empower them...it's...wonderful to get the patients involved directly in their care, and uh, inform them of the goals and the methods of achieving those goals.'

A few providers also explained that a necessary condition for the acceptability of this intervention was the 'well-established profile' and sometimes the 'cost-effectiveness' of thiazide diuretics:

'For hydrochlorothiazide, it is good...an enduring medication, a good medication...you just need the doctors to be aware of the effectiveness. But if you start promoting...all these fancy new medications [with this type of intervention]...I wouldn't encourage it.'

#### Negatives/reservations

Despite their overall receptivity to the patient activation approach, a number of providers expressed some concern or reservations about certain aspects of the intervention, a majority of which were focused on the use of incentives. Most reservations were expressed in the context of a positive opinion of the overall intervention strategy, as only one provider articulated a negative view of the intervention in general. Almost all the negatives/reservations expressed fit into two themes, with a third theme mentioned.

##### Financial incentives can create a conflict of interest

Four providers suggested the use of financial incentives created conflicting motivations for patients. A couple expressed this as a normative statement, suggesting simply that patients should be motivated not by money, but by what is good for their health; interestingly, a similar opinion was expressed by patients involved in the study. Two other providers suggested that the motivation created by the incentives could push patients to seek out a diuretic regardless of its suitability for them, thus compromising some of the provider's autonomy: 'If they are more interested in getting [the incentive], that kind of put pressure on us not to say no.'

A couple of providers also suggested that incentives may not be cost-effective, and one was concerned that patients might think the VA had an 'alternative motive' for offering an incentive because it is not typical practice at the VA.

However, it is worth noting that 13 of 17 providers asked actually had a positive or neutral view of the use of incentives. Most of these providers explained that if the incentives enhanced the patients' interest in their hypertension care, then they were fine with their inclusion, saying 'if it's going to work, I'm all for it.' Also, most providers said some patients seemed motivated by the $20 incentive to have a discussion, while providers felt few patients seemed motivated by the six-month co-pay reimbursement or pushed for a prescription because of it.

##### The intervention might undermine patient trust

Two providers expressed a concern that the intervention might suggest providers are giving inadequate care:

'As a physician...I often have a good reason for the decisions I make, and...I worry about it giving the message to, uh, the patient that 'your doctor should be doing this, and your doctor is not'.'

This concern was hypothetical for one provider, who also had a negative overall view of the intervention strategy. However, the other provider that expressed the concern did report a patient coming in with the impression that he received the letter because his provider had not prescribed the correct medication. This provider reported that the patient's concern was appeased in discussing the intervention further:

'I explained the situation to him...I told him why I didn't put him on hydrochlorothiazide, and why I would not put him on hydrochlorothiazide, and he was happy.'

This second provider had a positive view of the intervention, but was concerned that trust might still be undermined if a patient was not so easily appeased. It is worth noting that several other providers specifically volunteered that they didn't feel the intervention prompted any distrust:

'I did not have any...challenging interactions in the sense that somebody was either questioning my judgment, or upset, or thought there was an oversight...it was a very non-threatening conversation...and there wasn't any distrust, so they pretty well just believed my explanation if I said 'I don't think this is appropriate.' And they also, I didn't get the feeling of, you know, having them lose confidence in me if I said 'Yup...let's do it. Thanks for bring it to my attention.'

##### The wrong patients might be 'activated'

Similar to the previously described prescribing barrier--thiazides may not be a universally acceptable therapy--a couple of providers were also concerned that the intervention strategy might be targeted at patients that should not be on the promoted therapy. For example, one cautioned against targeting geriatric patients for thiazides, explaining that too often there are too many complications, and another explained if clinic rather than home blood pressure readings are used to identify target patients, it may create confusion in patients with controlled hypertension.

### Broader acceptability

In all, 18 of 20 providers asked had a positive opinion about using patient activation strategies on a broader basis for implementing hypertension or other therapy guidelines:

'I wouldn't mind seeing either more studies like this or even just having that be part of our practice of care...where the patient's getting letters...hypertension is a great idea or cholesterol would be another.'

As with explaining their opinions of the intervention itself, providers most often discussed how the patient activation strategy informs patients and facilitates discussions:

Interviewer: 'What do you think in general about promoting things such as new guideline therapies through patient-initiated interventions...taking information to the patient and having them bring it in?'

Provider one: 'I think that is actually a good idea...you can educate patient...and again...it make the job of physician easier, you know, when they come to the doctor they said, 'Is this right for me?' So then you don't have to start up the whole conversation again.'

Provider two: 'I think that's really kind of forming an alliance with your patient as, as you together determine what the best therapy is, so I don't, I don't see any problem with that. There's probably much to be gained.'

Provider three: 'I think that...would be...a wonderful idea, I think like I said earlier that, um, maybe prompting patients this way, uh, might...make them more interested and proactive with their healthcare.'

In explaining their opinion, other providers re-iterated the strategy had prompted useful changes in their management of some patients, and a few mentioned that they thought the strategy would prove cost-effective.

Two providers had negative or ambivalent views about using patient activation strategies on a larger scale. One supported broader use of the intervention to promote thiazides, but was hesitant to endorse its use for any other therapy, particularly for medications that were not as 'well-established' as thiazides. The other expressed concern that if the strategy was used for too many therapies, providers would quickly become saturated and the strategy would become ineffective.

### Sources that inform prescribing behavior

Through a number of questions providers listed sources that inform their prescribing behavior (Table 2). Since the intervention was focused on influencing their prescribing behavior, the list of sources offered insight into the providers' perceptions of other approaches to promoting evidence-based therapy. Most providers mentioned two or three sources, and few mentioned more than three. Most often mentioned was the scientific literature, although most of the nine providers that brought it up explained they don't have time to look at the literature regularly, or only look at a specific journal or two. Seven providers mentioned electronic databases, and other sources were more varied and disparate, each mentioned by five or fewer providers.

**Table 2:** Free-listed sources that inform provider prescribing behavior.*

Journals (9)	Peers (informally) (4)
Electronic Databases (7)	CME Lectures (3)
Websites (5)	Pharmacists (3)
Board Certification (4)	Residency/Fellowship (3)
Guideline Database (4)	Clinical Experience (3)
Opinion Leaders (3)	Institutional Memos/Directives (2)
Clinical Experience (3)	Grand Rounds (2)
Meetings (2)	Email Notifications (2)
Pharma Reps (2)	Medical School (1)

*Numbers in parentheses indicate the number of providers who mentioned the source

## Discussion

This patient activation intervention was not only effective at changing provider prescribing behavior [27], but was also acceptable to providers, most of whom had a positive opinion of both the intervention and the wider use of patient activation as an implementation strategy. In describing its efficacy, most providers focused first on the process of patient activation itself, describing how the intervention facilitated discussions by informing patients and making them more pro-active. Some described the effects of the intervention as similar to several other implementation strategies, acting as a reminder to consider a thiazide, flagging patients that were 'oversights,' or even prompting a re-evaluation of the evidence and rationale for prescribing thiazides as first-line therapy. Many also described the intervention as facilitating change in a manner more unique to patient activation, by 'empowering' patients and 'aligning' the 'priorities' of the patient and provider, with the consequence of making consultations more directed and efficient, or making patients more willing to accept a change in medications.

Uncontrolled hypertension may have been particularly well suited to this patient activation intervention and the ways providers described the intervention as facilitating change. Few providers indicated that the intervention provided them with any new information about thiazides, supporting previous evidence that the gap between evidence and practice in the case of hypertension management is more a matter of clinical inertia rather than provider knowledge [4,5,10,11]. These studies suggest that two primary contributors to clinical inertia--or failure to initiate or intensify therapy when indicated--may be clinical uncertainty and competing demands. It is possible that this intervention helped to overcome clinical uncertainty by providing a sort of confirmation that treatment would be appropriate, particularly for those cases in which providers described the intervention acting as a 'reminder' or highlighting 'oversights.' The targeted, personalized information contained in the letter, the presentation of the letter in clinical appointments, and the source of the letter could all have played a role in reinforcing for providers the certainty of the indication for treatment with thiazides. Further, providers' description of the intervention as 'aligning' patient and provider 'priorities' suggests the intervention reduced competing demands within the consultation, focusing the discussion on an asymptomatic condition that may otherwise be superseded by more acute or symptomatic concerns.

At the same time, some potential concerns about the process and acceptability of this intervention surround the patient-initiated approach to initiating changes in provider behavior. Patient-initiated demand for services often takes the form of specific requests, and such requests have been found to have a significant effect on providers' clinical decisions [40-42]. However, requests can consume limited consultation time and be perceived as demanding by physicians, while failure to fulfill a request, even when the requested service is not indicated, can threaten patient satisfaction and trust [40-42]. Of particular concern have been requests for potentially inappropriate prescribing or other improper or unnecessary care generated by the advertising techniques adopted for patient activation [33,43-50].

Interestingly, however, only one provider interviewed responded that a patient had specifically requested a thiazide prescription, and the vast majority instead described patients as initiating the discussion with a question about thiazides or presenting the intervention letter simply as a task they were to complete. Perhaps correspondingly, provider responses suggest there was little if any pressure to prescribe or sense of dissatisfaction or mistrust from patients if the provider decided a thiazide was not appropriate. A study of patient perspectives of the intervention found patients described their interactions with their providers in similar ways [34]. Given the efficacy of the intervention, it seems the letter and prompt for discussion preserved some of the positive influence that can be generated by a patient request without the pressure that could be viewed as negative. This suggests that, while the intervention was intended to create a specific demand for evidence-based therapy, there may be value in designing interventions that focus more on generating specific discussions rather than patient demand.

This idea is supported further by providers' comments on the value and acceptability of the intervention. Some did point out that it reinforced or broadened their utilization of thiazides as first-line therapy, but providers focused much more on the process, describing how they appreciated that the intervention facilitated discussions by informing patients and making them more pro-active while focusing the consultation by 'aligning' the priorities of the patient and provider. This emphasis on the interface itself suggests the effects or outcomes of this intervention are not limited to prescribing behavior, but rather include the provider-patient interaction generated by patient activation. Thus, even if patients were not prescribed for whatever reason, providers still valued the information patients received, the interest generated, and the discussions that were prompted.

This sort of informed patient participation has been increasingly advocated [50-56], and improved patient-provider concordance--or decision-making based on shared information and negotiation--may improve medication adherence and satisfaction for many patients [48,57]. Though providers emphasized the value of the discussions the intervention generated, the degree to which the prescribing decisions were shared in this case is not fully apparent from the interview data. The results do suggest that the satisfaction of providers with the discussions generated in this intervention is related at least in part to the selection of appropriately indicated patients and the focus provided by the intervention letter. Such targeted patient activation may prove more widely useful in both generating informed discussion and targeting it to improve patient-provider concordance.

While providers valued patient participation in this intervention, they did not look to patients as a source for new evidence to inform their prescribing behavior, as the absence of 'patients' from the free-listed sources in Table 2 illustrates. In describing the influence on their behavior, providers rather suggested the patients served as a reminder or reinforcement, while occasionally the letter itself provided new information or evidence considered by providers. However, the list of sources in Table 2 also illustrates that, even among providers in the same structured health system, sources that inform prescribing are disparate and variable. Yet, patients are one commonality with which all providers will interact, and through whom reinforcement of information can be directed. In combination with many providers' explanation that this intervention was particularly acceptable because thiazides are so well-established, this suggests patient activation as an implementation strategy is perhaps best suited for therapies for which the evidence-base is strong and widely disseminated, but which are nonetheless frequently overlooked, such as treatments for other common, chronic diseases or certain types of preventive care.

## Barriers

Several barriers were discussed by providers, the most frequent of which was particular characteristics of patients that may make them unsuitable for guideline therapy. The reasons given for this, such as age, co-morbidities, or contra-indications, are common and typically appropriate reasons for non-adherence to other guidelines [58,59]. In the case of hypertension, guidelines suggest thiazide diuretics as first-line therapy for uncomplicated hypertension, so it seems the autonomy of the provider to decide which patients could be classified as such was preserved.

## Negatives

Negatives were mostly expressed in the context of positive overall opinions of patient activation as an implementation strategy. Financial incentives were mentioned most often, though a majority of providers did have a positive or neutral opinion of using incentives. Interestingly, however, incentives may not even be necessary in this type of intervention. Discussion rates were high regardless of incentives, which showed only a modest effect.

While a few providers were concerned the intervention might undermine patients' trust in the quality of care they provide, only one reported a patient that was explicit about feeling this way, and this patient's concern was quickly allayed. This theme was only infrequently mentioned by patients as well [34]. Most providers emphasized that they welcomed the questions and discussion that were prompted, and several pointed out that patients were not accusatory or threatening in any way. Concerns about 'activating' the wrong patients reinforces that patients targeted for activation in future interventions should be carefully screened. However, with the autonomy of the providers seemingly intact in the intervention, they reported very few problems in letting patients know if they were not suitable for a thiazide diuretic.

## Limitations

There are several limitations to the study. First, its generalizability is limited due to the focus on VA providers from two VAMCs, as well as the small sample size. However, the qualitative design allowed for an information-rich analysis of provider perspectives of a patient activation strategy that could be expanded in future studies. Second, it relies on providers that agreed to be interviewed, and it is possible that such providers had more positive views of the intervention. Further, some providers may not have fully understood or remembered the intervention. The phone interviews were often conducted several months after providers saw patients, and several needed to be reminded about the details of the intervention. However, efforts were made during interviews to ensure providers were clear on the details and purpose of the intervention before giving their opinions, and most providers understood the intervention and remembered their consultations with little or no prompting or clarification. Finally, social desirability bias may have influenced both the providers and the interviewers. Providers may have reported that they understood and were guided by hypertension guidelines even if it is not clear they were. On the other hand, a social desirability bias may have hindered interviewers from explicitly asking providers why they were not prescribing thiaizides (even though they stated that they understood the guidelines). Such influences could have interfered with gaining a better understanding of why the discussion with patients prompted such an increase in prescribing.

## Summary

Patient activation was not only effective at implementing thiazide diuretics, but provider interviews suggested it was also acceptable in the context of this intervention, and could be similarly acceptable if utilized for broader implementation efforts. The effects on prescribing behavior were facilitated in some ways unique to patient activation, and providers did report valuing the changes in patient care prompted by the intervention, but they focused much more on the value of patient activation itself and the interest and discussions it generated. This emphasis suggests that the benefit of the intervention was not limited to its effects on prescribing behavior, but rather included facilitating a more mutually informed and focused clinical encounter.

Patient activation shows potential as an implementation strategy that may not only reinforce existing evidence or guidelines, but may also initiate and guide patient-provider discussions with the potential of 'aligning' the priorities of patients and providers. Patient activation should be tested as in implementation strategy in other areas of evidence-based medicine.

## Competing interests

The authors declare that they have no competing interests.

## Authors' contributions

CDB participated in the design of the interview guide, conducted interviews, performed and coordinated the qualitative analysis, and prepared the draft of the manuscript. MBW assisted with transcription and qualitative analysis and reviewed a draft of the manuscript. MVW and AJC contributed to the design of the study and reviewing and revising the manuscript. PJK was the principal investigator of the parent study and contributed significantly to the design of this study and conceptualizing, editing, and revising the manuscript. HSR oversaw the qualitative components of the parent study. For this paper, she coordinated the design of the study; conducted interviews; assisted in the analysis; and contributed significantly to conceptualizing, drafting, and revising the manuscript. All authors read and approved the final manuscript.

## Supplementary Material

Additional file 1 **Summative Evaluation for the VA Project to Implement Diuretics (VAPID)**. Interview guide developed to conduct semi-structured interviews with providers after completion of the intervention. Click here for file
