# Supplementary material for: Part II, Provider perspectives: should patients be activated to request evidence-based medicine? a qualitative study of the VA project to implement diuretics (VAPID)
Source: Implement Sci. 2010 Mar 18;5:24. doi: 10.1186/1748-5908-5-24 (PMC2856519; doi:10.1186/1748-5908-5-24)
Supplement: Additional file 1 — Summative Evaluation for the VA Project to Implement Diuretics (VAPID). Interview guide developed to conduct semi-structured interviews with providers after completion of the intervention. [file 1748-5908-5-24-S1.DOC]

**Additional File 1: Summative Evaluation for the VA Project to Implement Diuretics (VAPID)**

**(Implementing Evidence Based Treatment of Hypertension, P. Kaboli, PI)**

*During the Summative Evaluation process, consented provider participants (subjects) will be asked the following questions in an open-ended, semi-structured interview format. Additional questions (probes) may be asked to help clarify or elaborate on the following questions. At the beginning of the interview, participants will be reminded not to disclose any protected health information about their patients (i.e., names, birth dates, SSNs, etc.).*

This is __________________, and I'm here with participant number _________;

Today is ______ / ______ / _____.

Are you aware that you're being tape-recorded?

*_____Yes _____No*

Before we begin, I need to ask you to avoid using any HIPPA, or protected health information, about your patients.

1. Do you have any questions for me about the study or intervention before we begin?
2. Did you know about the study prior to the first time a patient brought in a letter and postcard to you?
3. What did you think about the intervention, sending an individualized letter to patients to bring to their provider?
4. Could you tell me your favorite story about a patient bringing in the letter?

**TYPICAL INTERACTION QUESTIONS (5-9)**

1. Please describe a typical interaction with a patient?

[possible probes] What did patients say? When did they bring it up? How long did the conversation take? How many patients of yours brought in letters? How many do you think you started on a thiazide? Did you take anyone off another hypertension medication and put them on a thiazide?

1. What do you think patients thought about the intervention? Did patients seem comfortable asking about being prescribed a thiazide?
2. Did patients tell you whether they wanted to be prescribed? How did this influence your decision to prescribe or not?
3. Did you ask patients whether they wanted to be prescribed a diuretic?
4. What did you think of the individualized patient information provided in the letter (last blood pressure reading, Framingham risk score, possible medications)? Did you use this information to talk with patients?

[possible probes] Did the patient seem to understand, then, the information from the letter, or the information being discussed?

1. Did the letter/postcard then change the conversations you had with your patients? How was your conversation different from your usual conversations with patients about hypertension?
2. What factors did you consider when you made your decision to prescribe a thiazide to a patient or not?
3. Did you tell patients the reasons for prescribing or not prescribing a thiazide? Why or why not?
4. Could you describe what sources of information you draw from when making these decisions? What sources inform your “hypertension practice”? [residency, trainings, board exams, grand rounds, guidelines, etc.]

Underlying this question is another question: what is the best way to disseminate new guideline therapy, evidence-based medicine, etc.?

1. Have you had a patient ask about a prescription medication they heard or read about in an ad? Was your experience talking with patients who brought in a letter similar to or different from those experiences? How so?

15. Do you think patients at the VA are more or less likely than patients in other settings to ask about new medications?

This ties into the previous question on your “hypertension practice”, but what sources of information do you refer to when a new medication come onto the market?

In our conversations with other providers, many spoke positively about the VA’s formulary and its emphasis on medications with established profiles. Do you agree with this sentiment? In your experience, what does it take for a medication to develop an established profile?

1. Patients were randomly assigned to one of three intervention groups. Two of the groups knew they could receive a $20 financial incentive if they discussed the information in the letter with you at their visit. If they had a co-pay, they could also receive a $48 reimbursement for their first six months of medication. Were you aware of this aspect of the study? *[Yes/No]*

[*If yes,*] do you think the incentive offers influenced your patients?

What led you to this conclusion?

Did they talk about the copay reimbursement? What did they say?

In your opinion, was the $20 incentive or co-pay reimbursement more influential?

1. What do you think generally about promoting new guideline therapies through patient-initiated interventions at the VA? Does the inclusion of an incentive change your opinion or the acceptability of such in intervention?
2. If the treatment guideline involved medications, what do you think about eliminating the co-pay for recommended medications? At the VA specifically?

[possible probes] Can you give me some examples of medications you think should be offered to patients without a co-pay? What would you think of a tiered co-pay system in the VA?

1. This is kind of an odd question, but do you think the letter changed the way you prescribed other patients, not just the ones that brought in a letter? Or the way you thought about the use of thiazides for hypertension?
2. Is there anything we should aware of? Do you have any examples of concerns/issues that arose because of the intervention?
3. Did I miss anything? Is there anything else about the intervention or study that you would like to add?
